# 4,9-Dioxa-1,3(1,2)-dibenzena-2(4,5)-1,3-oxazolidinacyclononaphane

**DOI:** 10.1107/S1600536810052517

**Published:** 2011-01-22

**Authors:** B. Balakrishnan, P. R. Seshadri, S. Purushothaman, R. Raghunathan

**Affiliations:** a Department of Physics, P. T. Lee Chengalvaraya Naicker College of Engineering & Technology, Kancheepuram 631 502, India; b PostGraduate & Research Department of Physics, Agurchand Manmull Jain College, Chennai 600 114, India; c Department of Organic Chemistry, University of Madras, Guindy Campus, Chennai 600 025, India

## Abstract

The oxazole ring in the title compound, C_20_H_23_NO_3_, adopts an envelope conformation while the 12-membered ring is in a chair conformation. The dihedral angle between the benzene rings is 37.8 (1)°. The crystal structure displays inter­molecular C—H⋯O hydrogen bonding.

## Related literature

For general background to cyclo­phanes and 1,3-dipolar cyclo­addition reactions, see: Whelligan *et al.* (2006 ▶); Poornachandran *et al.* (2008 ▶). For the chemistry of azomethine ylides, see; Longeon *et al.* (1990 ▶). For descriptions of ring conformations, see: Cremer & Pople (1975 ▶); Nardelli (1983 ▶).
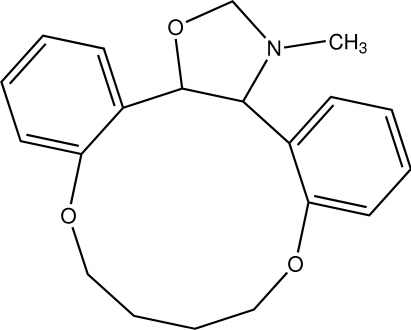


## Experimental

### 

#### Crystal data

C_20_H_23_NO_3_
*M* *_r_* = 325.39
Monoclinic, 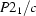

*a* = 9.5193 (7) Å
*b* = 13.0996 (8) Å
*c* = 13.6000 (9) Å
β = 96.704 (3)°
*V* = 1684.3 (2) Å^3^
*Z* = 4
Mo *K*α radiation
μ = 0.09 mm^−1^
*T* = 293 K
0.30 × 0.20 × 0.20 mm

#### Data collection

Bruker Kappa APEXII area-detector diffractometer
33618 measured reflections
3864 independent reflections
2937 reflections with *I* > 2σ(*I*)
*R*_int_ = 0.053

#### Refinement

*R*[*F* ^2^ > 2σ(*F* ^2^)] = 0.045
*wR*(*F* ^2^) = 0.146
*S* = 1.02
3864 reflections
218 parameters
H-atom parameters constrained
Δρ_max_ = 0.30 e Å^−3^
Δρ_min_ = −0.19 e Å^−3^

### 

Data collection: *APEX2* (Bruker, 2004 ▶); cell refinement: *SAINT* (Bruker, 2004 ▶); data reduction: *SAINT*; program(s) used to solve structure: *SHELXS97* (Sheldrick, 2008 ▶); program(s) used to refine structure: *SHELXL97* (Sheldrick, 2008 ▶); molecular graphics: *ORTEP-3* (Farrugia, 1997 ▶) and *PLATON* (Spek, 2009 ▶); software used to prepare material for publication: *SHELXL97* and *PLATON*.

## Supplementary Material

Crystal structure: contains datablocks I, global. DOI: 10.1107/S1600536810052517/bt5428sup1.cif

Structure factors: contains datablocks I. DOI: 10.1107/S1600536810052517/bt5428Isup2.hkl

Additional supplementary materials:  crystallographic information; 3D view; checkCIF report

## Figures and Tables

**Table 1:** Hydrogen-bond geometry (Å, °)

*D*—H⋯*A*	*D*—H	H⋯*A*	*D*⋯*A*	*D*—H⋯*A*
C14—H14⋯O1^i^	0.93	2.56	3.396 (2)	150

Symmetry code: (i) 
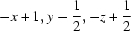
.

**Fig. 1.**
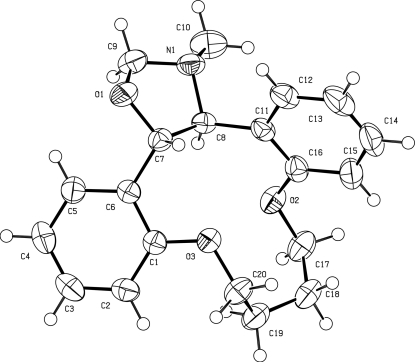
Molecular structure of the title compound, showing 30% probability displacement ellipsoids.

## supplementary crystallographic information

### Comment

Cyclophanes can act as a ligand in asymmetric catalysis (Whelligan *et al.*, 2006) and can as host molecules for the incorporation of guest molecules or ions. 1,3-dipolar cycloaddition (1,3-DC) reactions are efficient methods for the construction of heterocyclic units in a highly regio- and stereoselective manner (Poornachandran *et al.*, 2008). In particular the chemistry of azomethine ylides has gained importance in recent years as it serves as an expedient route for the construction of nitrogen heterocycles (Longeon *et al.*, 1990).

In the crystal structure of the title compound the oxazole ring is twisted along N1 - C9 and adopts an envelope conformation with the atom C9 displaced by -0.360 (0) \%A from the plane of the other ring atoms N1/O1//C7/C8. The puckering parameters (Cremer & Pople, 1975) and asymmetry parameters (Nardelli, 1983) are q~2~ =0.388 (2) Å, φ = 130.5 (2)°, Δ~S~ (C9) = 0.073 (1)° and Δ~2~ (C9) = 0.223 (1)°. In addition to van der Waals interactions the crystal structure is stabilized by C—H···O, hydrogen bonds.

### Experimental

A solution of O,*O*' coupled salicylaldehyde (bis aldehyde) using 1,4-dibromobutane (2 mmol) and sarcosine 2 (1 eq.) was refluxed in dry acetonitrile (20 ml) for about 6hrs under N2 atm. After the completion of reaction as indicated by TLC, acetonitrile was evaporated under reduced pressure.The crude product was purified by column chromatography using hexane: EtOAc (8:2) as eluent.

### Refinement

All H atoms were positioned geometrically and allowed to ride on their parent atoms, with C—H = 0.93–0.97 Å and Uĩso~(H) = 1.5*U*~eq~(C) for methylH atoms and 1.2*U*~eq~(C) for other H atoms.

### Crystal data

**Table:**

C_20_H_23_NO_3_	*F*(000) = 696
*M**_r_* = 325.39	*D*_x_ = 1.283 Mg m^−^^3^
Monoclinic, *P*2_1_/*c*	Mo *K*α radiation, λ = 0.71073 Å
*a* = 9.5193 (7) Å	Cell parameters from 5405 reflections
*b* = 13.0996 (8) Å	θ = 2.2–27.5°
*c* = 13.6000 (9) Å	µ = 0.09 mm^−^^1^
β = 96.704 (3)°	*T* = 293 K
*V* = 1684.3 (2) Å^3^	Block, colourless
*Z* = 4	0.30 × 0.20 × 0.20 mm

### Data collection

**Table:**

Bruker Kappa APEXII area-detector diffractometer	2937 reflections with *I* > 2σ(*I*)
Radiation source: fine-focus sealed tube	*R*_int_ = 0.053
graphite	θ_max_ = 27.5°, θ_min_ = 2.2°
ω scans	*h* = −12→12
33618 measured reflections	*k* = −17→11
3864 independent reflections	*l* = −17→17

### Refinement

**Table:**

Refinement on *F*^2^	Primary atom site location: structure-invariant direct methods
Least-squares matrix: full	Secondary atom site location: difference Fourier map
*R*[*F*^2^ > 2σ(*F*^2^)] = 0.045	Hydrogen site location: inferred from neighbouring sites
*wR*(*F*^2^) = 0.146	H-atom parameters constrained
*S* = 1.02	*w* = 1/[σ^2^(*F*_o_^2^) + (0.0761*P*)^2^ + 0.5036*P*] where *P* = (*F*_o_^2^ + 2*F*_c_^2^)/3
3864 reflections	(Δ/σ)_max_ < 0.001
218 parameters	Δρ_max_ = 0.30 e Å^−^^3^
0 restraints	Δρ_min_ = −0.19 e Å^−^^3^

### Special details

**Table:**

Geometry. All e.s.d.'s (except the e.s.d. in the dihedral angle between two l.s. planes) are estimated using the full covariance matrix. The cell e.s.d.'s are taken into account individually in the estimation of e.s.d.'s in distances, angles and torsion angles; correlations between e.s.d.'s in cell parameters are only used when they are defined by crystal symmetry. An approximate (isotropic) treatment of cell e.s.d.'s is used for estimating e.s.d.'s involving l.s. planes.
Refinement. Refinement of *F*^2^ against ALL reflections. The weighted *R*-factor *wR* and goodness of fit *S* are based on *F*^2^, conventional *R*-factors *R* are based on *F*, with *F* set to zero for negative *F*^2^. The threshold expression of *F*^2^ > σ(*F*^2^) is used only for calculating *R*-factors(gt) *etc*. and is not relevant to the choice of reflections for refinement. *R*-factors based on *F*^2^ are statistically about twice as large as those based on *F*, and *R*- factors based on ALL data will be even larger.

### Fractional atomic coordinates and isotropic or equivalent isotropic displacement parameters (Å^2^)

**Table:**

	*x*	*y*	*z*	*U*_iso_*/*U*_eq_	
C1	1.03220 (16)	0.21959 (11)	0.36089 (11)	0.0333 (3)	
C2	1.17864 (17)	0.22294 (13)	0.37457 (13)	0.0425 (4)	
H2	1.2308	0.1756	0.3431	0.051*	
C3	1.24706 (18)	0.29660 (15)	0.43489 (15)	0.0501 (4)	
H3	1.3454	0.2989	0.4436	0.060*	
C4	1.17130 (19)	0.36634 (14)	0.48207 (14)	0.0507 (5)	
H4	1.2177	0.4161	0.5224	0.061*	
C5	1.02488 (18)	0.36210 (13)	0.46926 (13)	0.0420 (4)	
H5	0.9736	0.4091	0.5019	0.050*	
C6	0.95331 (15)	0.28944 (11)	0.40890 (11)	0.0324 (3)	
C7	0.79349 (16)	0.28849 (11)	0.39086 (12)	0.0347 (3)	
H7	0.7647	0.2910	0.3193	0.042*	
C8	0.72211 (15)	0.19497 (11)	0.43422 (11)	0.0328 (3)	
H8	0.7910	0.1597	0.4817	0.039*	
C9	0.6768 (2)	0.33945 (15)	0.51895 (15)	0.0530 (5)	
H9A	0.6059	0.3865	0.5377	0.064*	
H9B	0.7489	0.3302	0.5748	0.064*	
C10	0.5729 (2)	0.18140 (19)	0.56714 (16)	0.0649 (6)	
H10A	0.6546	0.1667	0.6133	0.097*	
H10B	0.5324	0.1187	0.5406	0.097*	
H10C	0.5045	0.2175	0.6004	0.097*	
C11	0.65494 (15)	0.11940 (12)	0.35916 (11)	0.0353 (3)	
C12	0.53468 (18)	0.14587 (15)	0.29742 (13)	0.0479 (4)	
H12	0.4966	0.2109	0.3018	0.058*	
C13	0.46994 (19)	0.0771 (2)	0.22916 (15)	0.0600 (6)	
H13	0.3890	0.0958	0.1881	0.072*	
C14	0.5258 (2)	−0.01856 (18)	0.22248 (15)	0.0601 (6)	
H14	0.4822	−0.0649	0.1768	0.072*	
C15	0.6455 (2)	−0.04689 (15)	0.28250 (14)	0.0511 (5)	
H15	0.6830	−0.1120	0.2772	0.061*	
C16	0.71063 (17)	0.02175 (12)	0.35126 (12)	0.0381 (4)	
C17	0.9048 (2)	−0.09245 (13)	0.40456 (14)	0.0490 (4)	
H17A	0.8388	−0.1491	0.4015	0.059*	
H17B	0.9719	−0.1016	0.4633	0.059*	
C18	0.9838 (2)	−0.09675 (14)	0.31489 (14)	0.0507 (5)	
H18A	0.9151	−0.0983	0.2564	0.061*	
H18B	1.0360	−0.1605	0.3168	0.061*	
C19	1.0867 (2)	−0.01005 (13)	0.30337 (14)	0.0503 (4)	
H19A	1.1248	0.0131	0.3688	0.060*	
H19B	1.1650	−0.0367	0.2714	0.060*	
C20	1.0258 (2)	0.08130 (14)	0.24472 (13)	0.0482 (4)	
H20A	1.1022	0.1162	0.2172	0.058*	
H20B	0.9604	0.0569	0.1897	0.058*	
N1	0.61418 (14)	0.24377 (11)	0.48733 (10)	0.0425 (3)	
O1	0.73653 (13)	0.37542 (9)	0.43567 (10)	0.0488 (3)	
O2	0.82817 (12)	0.00023 (8)	0.41494 (8)	0.0418 (3)	
O3	0.95377 (12)	0.15360 (8)	0.29970 (9)	0.0398 (3)	

### Atomic displacement parameters (Å^2^)

**Table:**

	*U*^11^	*U*^22^	*U*^33^	*U*^12^	*U*^13^	*U*^23^
C1	0.0358 (8)	0.0314 (7)	0.0331 (7)	−0.0014 (6)	0.0058 (6)	0.0058 (6)
C2	0.0354 (8)	0.0427 (9)	0.0509 (10)	0.0030 (7)	0.0114 (7)	0.0064 (7)
C3	0.0323 (8)	0.0550 (11)	0.0620 (11)	−0.0054 (7)	0.0012 (8)	0.0121 (9)
C4	0.0467 (10)	0.0496 (10)	0.0527 (11)	−0.0123 (8)	−0.0067 (8)	−0.0007 (8)
C5	0.0433 (9)	0.0390 (9)	0.0431 (9)	−0.0012 (7)	0.0021 (7)	−0.0025 (7)
C6	0.0322 (7)	0.0319 (7)	0.0333 (7)	−0.0001 (6)	0.0042 (6)	0.0055 (6)
C7	0.0344 (8)	0.0326 (7)	0.0367 (8)	0.0047 (6)	0.0026 (6)	0.0009 (6)
C8	0.0291 (7)	0.0375 (8)	0.0318 (7)	0.0037 (6)	0.0042 (6)	0.0007 (6)
C9	0.0487 (10)	0.0550 (11)	0.0568 (11)	0.0091 (8)	0.0124 (8)	−0.0136 (9)
C10	0.0673 (13)	0.0814 (15)	0.0513 (12)	0.0039 (11)	0.0287 (10)	0.0040 (10)
C11	0.0303 (7)	0.0439 (8)	0.0325 (8)	−0.0055 (6)	0.0071 (6)	0.0007 (6)
C12	0.0339 (8)	0.0651 (12)	0.0442 (9)	−0.0007 (8)	0.0023 (7)	−0.0005 (8)
C13	0.0363 (9)	0.0961 (17)	0.0455 (10)	−0.0113 (10)	−0.0039 (8)	−0.0031 (10)
C14	0.0527 (11)	0.0779 (15)	0.0492 (11)	−0.0288 (10)	0.0047 (9)	−0.0154 (10)
C15	0.0552 (11)	0.0491 (10)	0.0496 (10)	−0.0175 (8)	0.0088 (8)	−0.0093 (8)
C16	0.0405 (8)	0.0405 (8)	0.0340 (8)	−0.0097 (6)	0.0078 (6)	0.0005 (6)
C17	0.0680 (12)	0.0342 (9)	0.0441 (9)	0.0083 (8)	0.0040 (8)	0.0021 (7)
C18	0.0648 (12)	0.0383 (9)	0.0485 (10)	0.0084 (8)	0.0043 (9)	−0.0104 (7)
C19	0.0559 (11)	0.0435 (10)	0.0518 (10)	0.0095 (8)	0.0071 (8)	−0.0102 (8)
C20	0.0598 (11)	0.0477 (10)	0.0387 (9)	0.0055 (8)	0.0124 (8)	−0.0061 (7)
N1	0.0366 (7)	0.0511 (8)	0.0415 (8)	0.0051 (6)	0.0124 (6)	−0.0044 (6)
O1	0.0459 (7)	0.0345 (6)	0.0667 (8)	0.0107 (5)	0.0093 (6)	−0.0020 (5)
O2	0.0524 (7)	0.0364 (6)	0.0357 (6)	0.0058 (5)	0.0010 (5)	−0.0028 (5)
O3	0.0401 (6)	0.0356 (6)	0.0442 (6)	0.0002 (4)	0.0069 (5)	−0.0042 (5)

### Geometric parameters (Å, °)

**Table:**

C1—O3	1.3610 (19)	C10—H10C	0.9600
C1—C2	1.385 (2)	C11—C12	1.382 (2)
C1—C6	1.394 (2)	C11—C16	1.394 (2)
C2—C3	1.380 (3)	C12—C13	1.385 (3)
C2—H2	0.9300	C12—H12	0.9300
C3—C4	1.369 (3)	C13—C14	1.368 (3)
C3—H3	0.9300	C13—H13	0.9300
C4—C5	1.385 (2)	C14—C15	1.373 (3)
C4—H4	0.9300	C14—H14	0.9300
C5—C6	1.383 (2)	C15—C16	1.390 (2)
C5—H5	0.9300	C15—H15	0.9300
C6—C7	1.513 (2)	C16—O2	1.362 (2)
C7—O1	1.4281 (18)	C17—O2	1.432 (2)
C7—C8	1.550 (2)	C17—C18	1.507 (3)
C7—H7	0.9800	C17—H17A	0.9700
C8—N1	1.4694 (19)	C17—H17B	0.9700
C8—C11	1.509 (2)	C18—C19	1.520 (3)
C8—H8	0.9800	C18—H18A	0.9700
C9—O1	1.406 (2)	C18—H18B	0.9700
C9—N1	1.432 (2)	C19—C20	1.515 (3)
C9—H9A	0.9700	C19—H19A	0.9700
C9—H9B	0.9700	C19—H19B	0.9700
C10—N1	1.449 (2)	C20—O3	1.430 (2)
C10—H10A	0.9600	C20—H20A	0.9700
C10—H10B	0.9600	C20—H20B	0.9700
			
O3—C1—C2	125.02 (14)	C11—C12—C13	121.06 (19)
O3—C1—C6	114.61 (13)	C11—C12—H12	119.5
C2—C1—C6	120.33 (14)	C13—C12—H12	119.5
C3—C2—C1	119.99 (16)	C14—C13—C12	119.61 (18)
C3—C2—H2	120.0	C14—C13—H13	120.2
C1—C2—H2	120.0	C12—C13—H13	120.2
C4—C3—C2	120.49 (16)	C13—C14—C15	120.65 (18)
C4—C3—H3	119.8	C13—C14—H14	119.7
C2—C3—H3	119.8	C15—C14—H14	119.7
C3—C4—C5	119.45 (16)	C14—C15—C16	119.94 (19)
C3—C4—H4	120.3	C14—C15—H15	120.0
C5—C4—H4	120.3	C16—C15—H15	120.0
C6—C5—C4	121.39 (16)	O2—C16—C15	124.24 (16)
C6—C5—H5	119.3	O2—C16—C11	115.64 (13)
C4—C5—H5	119.3	C15—C16—C11	120.12 (16)
C5—C6—C1	118.35 (14)	O2—C17—C18	114.82 (14)
C5—C6—C7	121.23 (14)	O2—C17—H17A	108.6
C1—C6—C7	120.36 (13)	C18—C17—H17A	108.6
O1—C7—C6	110.43 (12)	O2—C17—H17B	108.6
O1—C7—C8	105.22 (12)	C18—C17—H17B	108.6
C6—C7—C8	114.96 (12)	H17A—C17—H17B	107.5
O1—C7—H7	108.7	C17—C18—C19	116.37 (15)
C6—C7—H7	108.7	C17—C18—H18A	108.2
C8—C7—H7	108.7	C19—C18—H18A	108.2
N1—C8—C11	110.64 (12)	C17—C18—H18B	108.2
N1—C8—C7	101.88 (12)	C19—C18—H18B	108.2
C11—C8—C7	115.52 (12)	H18A—C18—H18B	107.3
N1—C8—H8	109.5	C20—C19—C18	115.66 (16)
C11—C8—H8	109.5	C20—C19—H19A	108.4
C7—C8—H8	109.5	C18—C19—H19A	108.4
O1—C9—N1	104.16 (14)	C20—C19—H19B	108.4
O1—C9—H9A	110.9	C18—C19—H19B	108.4
N1—C9—H9A	110.9	H19A—C19—H19B	107.4
O1—C9—H9B	110.9	O3—C20—C19	115.15 (14)
N1—C9—H9B	110.9	O3—C20—H20A	108.5
H9A—C9—H9B	108.9	C19—C20—H20A	108.5
N1—C10—H10A	109.5	O3—C20—H20B	108.5
N1—C10—H10B	109.5	C19—C20—H20B	108.5
H10A—C10—H10B	109.5	H20A—C20—H20B	107.5
N1—C10—H10C	109.5	C9—N1—C10	114.09 (16)
H10A—C10—H10C	109.5	C9—N1—C8	103.75 (13)
H10B—C10—H10C	109.5	C10—N1—C8	112.89 (15)
C12—C11—C16	118.61 (15)	C9—O1—C7	106.60 (12)
C12—C11—C8	120.11 (15)	C16—O2—C17	120.20 (13)
C16—C11—C8	121.28 (14)	C1—O3—C20	118.54 (13)
			
O3—C1—C2—C3	176.77 (15)	C12—C13—C14—C15	−0.2 (3)
C6—C1—C2—C3	−0.9 (2)	C13—C14—C15—C16	0.4 (3)
C1—C2—C3—C4	0.4 (3)	C14—C15—C16—O2	178.93 (16)
C2—C3—C4—C5	0.4 (3)	C14—C15—C16—C11	−0.2 (3)
C3—C4—C5—C6	−0.7 (3)	C12—C11—C16—O2	−179.29 (14)
C4—C5—C6—C1	0.2 (2)	C8—C11—C16—O2	−0.2 (2)
C4—C5—C6—C7	−176.75 (15)	C12—C11—C16—C15	−0.1 (2)
O3—C1—C6—C5	−177.30 (13)	C8—C11—C16—C15	179.04 (15)
C2—C1—C6—C5	0.6 (2)	O2—C17—C18—C19	54.9 (2)
O3—C1—C6—C7	−0.3 (2)	C17—C18—C19—C20	−90.5 (2)
C2—C1—C6—C7	177.57 (14)	C18—C19—C20—O3	83.15 (19)
C5—C6—C7—O1	5.6 (2)	O1—C9—N1—C10	167.06 (15)
C1—C6—C7—O1	−171.33 (13)	O1—C9—N1—C8	43.82 (17)
C5—C6—C7—C8	−113.29 (16)	C11—C8—N1—C9	−154.70 (14)
C1—C6—C7—C8	69.83 (18)	C7—C8—N1—C9	−31.35 (16)
O1—C7—C8—N1	8.90 (15)	C11—C8—N1—C10	81.28 (18)
C6—C7—C8—N1	130.61 (13)	C7—C8—N1—C10	−155.38 (15)
O1—C7—C8—C11	128.87 (13)	N1—C9—O1—C7	−38.20 (17)
C6—C7—C8—C11	−109.42 (15)	C6—C7—O1—C9	−107.21 (15)
N1—C8—C11—C12	45.81 (19)	C8—C7—O1—C9	17.41 (16)
C7—C8—C11—C12	−69.25 (18)	C15—C16—O2—C17	9.1 (2)
N1—C8—C11—C16	−133.28 (15)	C11—C16—O2—C17	−171.70 (14)
C7—C8—C11—C16	111.66 (15)	C18—C17—O2—C16	70.1 (2)
C16—C11—C12—C13	0.2 (2)	C2—C1—O3—C20	0.2 (2)
C8—C11—C12—C13	−178.90 (16)	C6—C1—O3—C20	177.96 (13)
C11—C12—C13—C14	−0.1 (3)	C19—C20—O3—C1	77.49 (19)

### Hydrogen-bond geometry (Å, °)

**Table:**

*D*—H···*A*	*D*—H	H···*A*	*D*···*A*	*D*—H···*A*
C5—H5···O1	0.93	2.37	2.735 (2)	103
C8—H8···O2	0.98	2.32	2.767 (2)	107
C14—H14···O1^i^	0.93	2.56	3.396 (2)	150

Symmetry codes: (i) −*x*+1, *y*−1/2, −*z*+1/2.
